# A 77-dB Dynamic-Range Analog Front-End for Fine-Dust Detection Systems with Dual-Mode Ultra-Low Noise TIA †

**DOI:** 10.3390/s21196360

**Published:** 2021-09-23

**Authors:** Reza E. Rad, Arash Hejazi, Seyed-Ali H. Asl, Khuram Shehzad, Deeksha Verma, SungJin Kim, Behnam S. Rikan, YoungGun Pu, Joon Tae Kim, Keum Cheol Hwang, Youngoo Yang, Kang-Yoon Lee

**Affiliations:** 1 Department of Electrical and Computer Engineering, Sungkyunkwan University, Suwon 16419, Korea; reza@skku.edu (R.E.R.); arash@skku.edu (A.H.); saha@skku.edu (S.-A.H.A.); khuram1698@skku.edu (K.S.); deeksha27@skku.edu (D.V.); sun107ksj@skku.edu (S.K.); behnam@skku.edu (B.S.R.); hara1015@skku.edu (Y.P.); khwang@skku.edu (K.C.H.); yang09@skku.edu (Y.Y.); 2 SKAIChips Co., Ltd., Suwon 16419, Korea; 3 Department of Electrical and Electronic Engineering, Konkuk University, Seoul 05029, Korea; jtkim@konkuk.ac.kr

**Keywords:** dual-mode gain, ultra-low noise, transimpedance amplifier, TIA, High-Gain, fine-dust detection, analog front-end

## Abstract

This paper presents an analog front-end for fine-dust detection systems with a 77-dB-wide dynamic range and a dual-mode ultra-low noise TIA with 142-dBΩ towards the maximum gain. The required high sensitivity of the analog signal conditioning path dictates having a high sensitivity at the front-end while the Input-Referred Noise (IRN) is kept low. Therefore, a TIA with a high sensitivity to detected current bio-signals is provided by a photodiode module. The analog front end is formed by the TIA, a DC-Offset Cancellation (DCOC) circuit, a Single-to-Differential Amplifier (SDA), and two Programmable Gain Amplifiers (PGAs). Gain adjustment is implemented by a coarse-gain-step using selective loads with four different gain values and fine-gain steps by 42 dB dynamic range during 16 fine steps. The settling time of the TIA is compensated using a capacitive compensation which is applied for the last stage. An off-state circuitry is proposed to avoid any off-current leakage. This TIA is designed in a 0.18 µm standard CMOS technology. Post-layout simulations show a high gain operation with a 67 dB dynamic range, input-referred noise, less than 600 fA/√Hz in low frequencies, and less than 27 fA/√Hz at 20 kHz, a minimum detectable current signal of 4 pA, and a 2.71 mW power consumption. After measuring the full path of the analog signal conditioning path, the experimental results of the fabricated chip show a maximum gain of 142 dB for the TIA. The Single-to-Differential Amplifier delivers a differential waveform with a unity gain. The PGA1 and PGA2 show a maximum gain of 6.7 dB and 6.3 dB, respectively. The full-path analog front-end shows a wide dynamic range of up to 77 dB in the measurement results.

## 1. Introduction

Recently, air pollution has been a major concern. Based on the recent World Health Organization (WHO) estimations, air pollution is attributing directly to the death of seven million people every year [1]. Ambient air pollution (outdoor) and household air pollution are the major causes of diseases and premature death and are estimated to have a death rate of 4.2 and 3.8 million deaths per year, respectively [1]. The motivation of several studies on air pollution-detecting technologies is to record accurate data on the density of air pollution. Fine-dust air pollution has recently been considered one of the most harmful air pollution types [1]. Fine particulate matter or PM2.5 refers to the particles in the air with a size of less than 2.5 µm [2]. Several dangerous health issues such as birth defects, lung cancer, asthma, and premature death are reported as the side-effects of the PM2.5 particles [3,4,5]. Various studies have been done to estimate air pollution in both indoor and outdoor applications [6,7,8,9]. In [6,7], urban fine dust monitoring was proposed. In [8], an unmanned vehicle was proposed to collect the information. In [9], research was based on the analysis of the captured images through the satellites from the atmosphere. In this paper, an analog front-end was proposed for a fine-dust detection system with high sensitivity and gain adjustability to perform the signal conditioning task over the input bio-signal from the sensor.

## 2. The Proposed Fine-Dust Detection Analog Front-End Signal Conditioning Path

A fine-dust detection system Integrated Circuit (IC) must be capable of sensing the density and variations in fine-dust particles through an externally-placed sensor. Currently, the sensors are formed based on Photo Diode (PD) modules. A weak signal will be fed to the IC through the sensor. The received signal will be delivered to the Analog Front-End (AFE) of the system inside the IC to perform an analog signal conditioning process and provide a well-prepared analog signal to be converted to a digital signal for additional processes.

**Figure 1:**
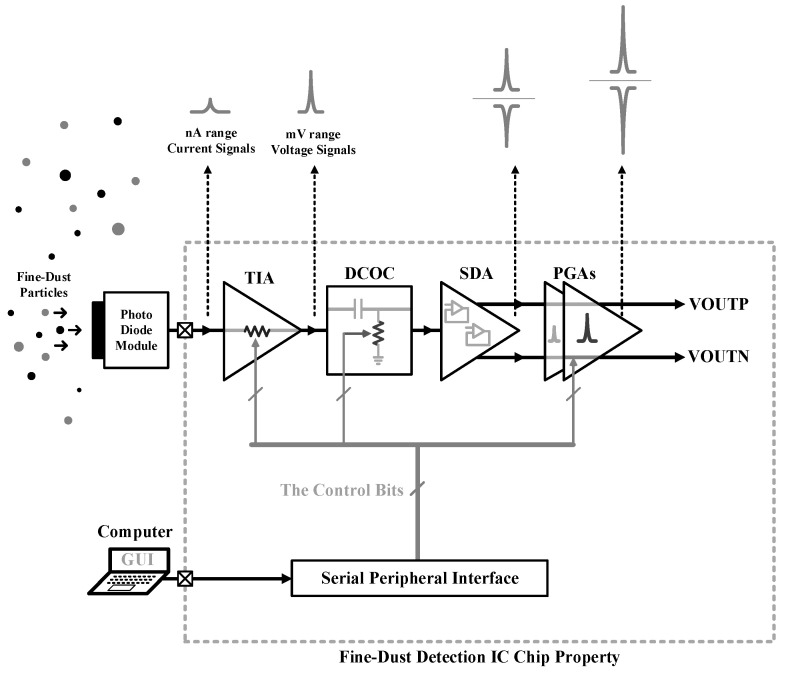
The top block-diagram of the fine-dust detection system IC.

The AFE path is designed to perform the signal conditioning process through a TIA as the first stage of the AFE chain, which provides a controlled signal amplification. A DC-Offset Cancellation (DCOC) circuit is placed after the TIA to adjust the output common-mode of the TIA. A Single-to-Differential Amplifier (SDA) makes a differential copy of the signal. Then, two Programmable Gain Amplifier (PGA) blocks are placed to shape the final version of the processed analog signal and satisfy the full-scale voltage of the analog-to-digital conversion. The proposed analog front-end is shown in Figure 1. A Serial Peripheral Interface (SPI) digital controller plays the role of digital controller of the chip through an SPI programmer in a computer.

## 3. Dual-Mode Adjustable Gain Low Noise TIA with Indirect Feedback Biasing

A sensor module which is formed by a photodiode (PD) and a laser beam, plays the role of air particle detection. The sensor generates weak current signals proportional to the passing dust particles. The passing particles through the module change the observed laser light by the PD. Therefore, the density and movement of the fine-dust particles will be detected through the current variations of the PD. These particles cause instantaneous signal variations in low frequencies (20 kHz). This signal in terms of current contains nano-ampere range values, and each value must be quantized to the specific data. To perform well-controlled flexible signal conditioning, a controlled amplification with high gain within a limited bandwidth is demanding. The obtained signal is noisy, which makes the Input Referred Noise (IRN) of the first stage of the signal conditioning AFE circuit critical. Due to the low-frequency input signals, the challenging aspects of the design of the TIA are the IRN and transimpedance gain.

The proposed fine-dust detection system features gain controllability of the TIA with fine- and coarse-gain steps, which enable the controller to perform a dual-mode AGC for the TIA through the SPI digital controller. The light current condition may occur for the operation of the PD and produce higher amplitudes, which cause the TIA to experience high currents in the micro-ampere range. This phenomenon is suppressed through the adjustable gain mechanisms to increase the dynamic input range of the TIA and improve the sensitivity of the TIA, consequently.

Even though a DCOC block is placed between the TIA and SDA, it is preferred to have a fixed common voltage at the output node of the TIA when the gain is changing. Common mode variations of the output node of TIA cause many drawbacks for the operation of the next stages and limit the reliability of the TIA to the minimum difference of the output common-mode voltage and the linear operating over-drive voltage of the output stage transistors. Therefore, bias-based gain adjustments that change the common mode of the output node are not suggested.

**Figure 2:**
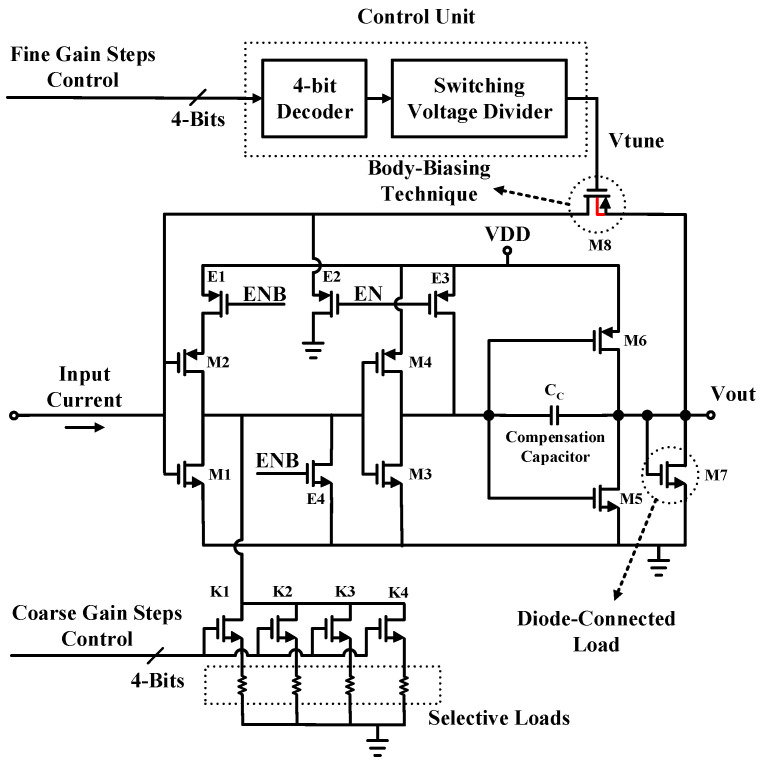
The proposed dual-gain-mode TIA.

The top structure of the proposed novel TIA is illustrated in Figure 2. The core of the TIA is formed by three push-pull inverting amplifiers, which have been well examined previously [10,11]. The core of the TIA is modified to involve standby circuitry for portability aid which results in an almost zero off-current. This happens through the proposed circuitry implemented by the E1, E2, E3, and E4 ENB switches for the TIA structure, which causes trouble with having three push-pull inverting amplifiers in series, and resists going into standby mode.

The noise contribution of the circuitry is considered in a design reaching a long channel length for the E2 P-MOS transistor with a large enough width with many fingers. The same goes for the E4 N-MOS transistor with a smaller width and length in comparison with the E2, but for E1 and E3 P-MOS transistors, a design with minimum length, and a width three times larger than the length, is enough.

The gain stages of the TIA, including the M1-M2, M3-M4, and M5-M6 pairs, do not take advantage of any bias circuit; therefore, their overdrive voltages are restricted by their ratios. In this way, the common voltage, before adding M7, is restricted to 1.1 V due to the transistor ratios, resulting in a minimized IRN. After adding M7, this value is decreased to 1 V, while the 1/gm impedance seen at the drain of the M7 to the ground acts as a fixed initial load and provides more reliable control over the feedback resistor through M8.

A compensation capacitor (Cc) is used to enhance a better settling time for the TIA when a 60-degree phase margin is obtained. The gain adjustment mechanism of the proposed structure is implemented through two parallel operation modes with coarse-gain steps and fine-gain steps [12].

The coarse-gain-step mechanism involves four selective loads, which are switched by K1 to K4 switches, controlled by SPI. Therefore, four coarse-gain states are provided by the proposed coarse-gain-step mechanism. SPI picks a load from the four options through an only-ON switch order. This implementation proposes gain adjustability by not touching input and output branches to avoid the mentioned loading issues.

**Figure 3:**
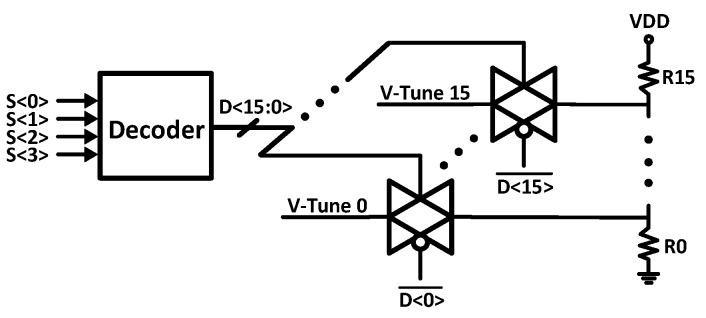
The fine-gain step bias trimming circuit.

Furthermore, the output common mode of the TIA is not varying during load switching. The fine-gain step mechanism is controlled by the gate voltage of M8 as the feedback transistor. The gate voltage is controlled through a decoder-based circuit in Figure 3. The circuit creates 16 individual values with small fine steps. Moreover, the M8 transistor is designed using the body-biasing technique, which results in a considerable reduction of the total IRN. The reason for the dual-mode gain adjustability of the TIA is to implement the hardware with a wide vision in terms of the controller and data analyzer software.

Gain modes of the TIA are controlled based on an Indirect Operating Point Biasing (IOPB) technique. As is discussed in [10,11], the gain of a TIA with such a structure can change by changing the bias voltage of the feedback transistor. The importance of the fully digitally controlled amplifier is evident when the target is a well-controlled flexible hardware implementation, following various controller software scenarios. Therefore, fully digitally controlled systems implementation results in a multiplication of the number of coarse- and fine-gain steps. In terms of circuit design, normally, it reaches a non-optimized complex design full of switches and passive elements to control the bias of the M8 transistor in addition to selective-load circuitry.

**Figure 4:**
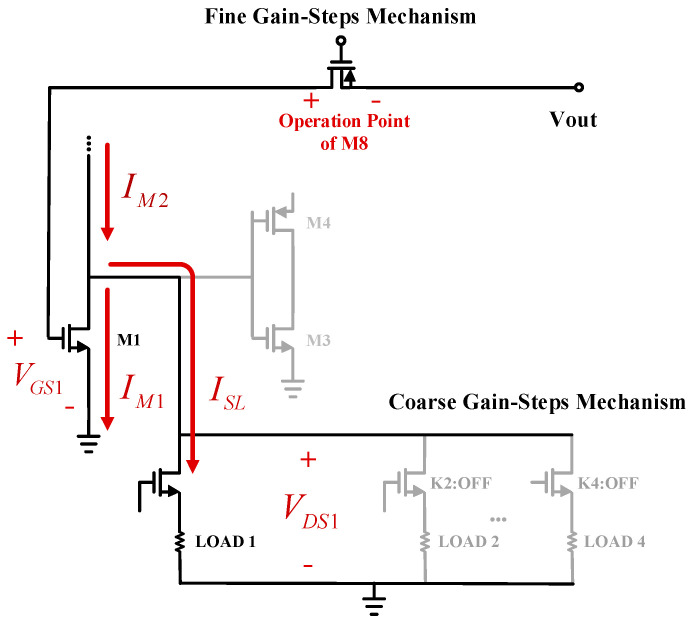
The gain-selection mechanism of the proposed TIA.

In the design of the proposed TIA, complexity is handled using the IOPB technique, which has a huge enhancement in terms of controllability enhancement, area occupation, and power consumption. Using the IOPB technique, when the selective load switches to another (as a coarse-gain-step change), the operating point bias of the M8 also changes to a different operating point indirectly. Therefore, the fine adjustment occurs around the operation node due to the selected load through the gate voltage of the M8. The IOPB technique can be tracked as follow:

(1) $$I_{\mathrm{SL}}=\frac{V_{\mathrm{DS}1}}{R_{\mathrm{SL}}}$$

where $I_{\mathrm{SL}}$ and $V_{\mathrm{SL}}$ are the DC current and DC voltage of the picked selective load. This is shown in Figure 4. By switching among the loads, the current of the load branch varies, which changes the current of the M1 as follow:

(2) $$I_{M1}=I_{M2}-I_{\mathrm{SL}}$$

in this way, by switching among the selective loads, the current of the M1 changes when the current of M2 stays fixed, while $V_{\mathrm{DS}1}$ and $V_{\mathrm{TH}1}$ also stay fixed. Therefore, following the exponential current formula, it can be shown:

(3) $$I_{M1}=\frac{1}{2}\mu_{n1}C_{\mathrm{ox}1}\frac{W_{1}}{L_{1}}\left(V_{\mathrm{GS}1}-V_{\mathrm{TH}1}\right)\left(1+{\lambda V}_{\mathrm{DS}}\right)$$

since all the parameters are kept fixed, else $V_{\mathrm{GS}1}$. Therefore, the gate voltage of M1 changes when $I_{M1}$ varies, due to the selected load, to satisfy the current equation. On the other hand, variations in gate voltage of the M1 reflect the operating point of the M8. In conclusion, the operation point of the M8 is controlled indirectly through the selective loads as the IOPB technique. Despite the coarse-gain-step mechanism, for the fine-gain step mechanism, the operating point is adjusted with direct biasing through the gate of the M8.

On the other hand, the gain of the first stage, which is directly controlled through the selective loads, is adjusted by the transconductance of the selective loads as below:

(4) $$A_{1}=\frac{-\left(g_{m1}+g_{m2}\right)}{g_{\mathrm{ds}1}+g_{\mathrm{ds}2}+g_{L}}$$

where the transconductances of $g_{m1}$, $g_{m2}$, and the channel transconductances of $g_{\mathrm{ds}1}$ and $g_{\mathrm{ds}2}$ are related to the M1 and M2, respectively. The $g_{L}$ is controlled by selective loads. The pole of the first stage, which mainly affects the bandwidth, is known in (3) where $C_{\mathrm{gd}1,2}$ and $C_{\mathrm{bd}1,2}$ are parasitic capacitances of M1 and M2.

(5) $$P_{1}=\frac{-\left(g_{\mathrm{ds}1}+g_{\mathrm{ds}2}+g_{L}\right)}{C_{\mathrm{gd}1}+C_{\mathrm{gd}2}+C_{\mathrm{bd}1}+C_{\mathrm{bd}2}}$$

Finally, stability is concerned for the minimum and maximum-gain settings of the TIA through the phase and gain margins.

## 4. DC Offset Cancellation (DCOC)

**Figure 5:**
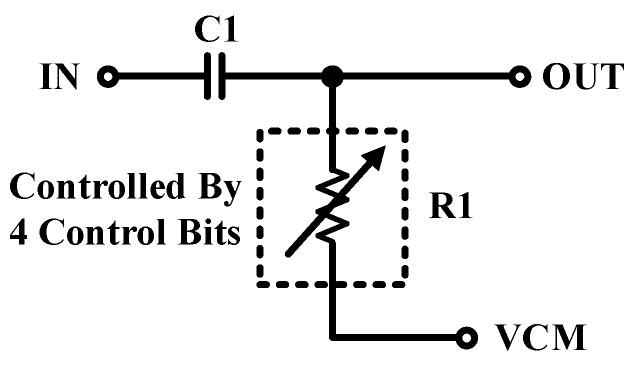
The schematic of the DCOC block of the system.

A DCOC block is placed between TIA and DSA blocks to match the signal path in terms of the common-mode voltage of the signal. The circuit of the DCOC block is shown in Figure 5. An adjustment control on the common-mode level is performed digitally. This controllability is performed through four control bits.

## 5. Single-to-Differential Amplifier (SDA)

**Figure 6:**
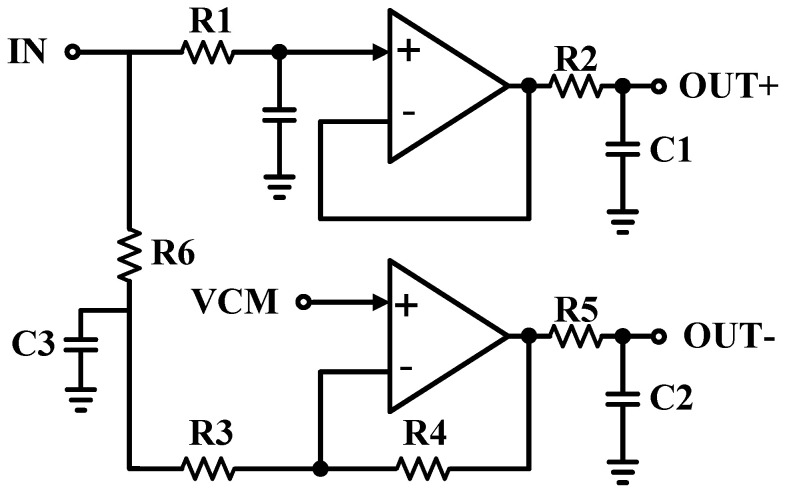
The schematic of the SDA of the system.

To convert the signal from the single to differential form, the SDA was designed, as shown in Figure 6. The SDA is placed after TIA and DCOC blocks in the AFE chain. The used structure is formed based on two-stage Operational Amplifiers (Op-Amps). VCM comes from the DCOC.

## 6. Programmable Gain Amplifier (PGA)

**Figure 7:**
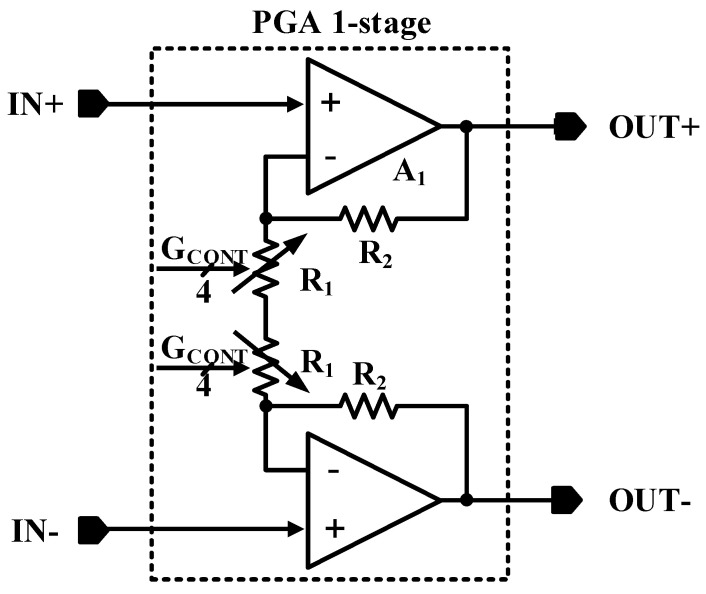
The schematic of the PGAs of the system.

To perform additional well-controlled amplification on the converted signal from single-ended to differential by the SDA, two PGA stages are used as the last signal conditioning blocks of the AFE chain. Figure 7 shows the PGA block diagram, which is designed using two similar op-amps and the circuitry, providing a programmable gain amplification of 1+(R_2⁄R_1) while R_1 is adjustable digitally. Controllability of the PGA is provided through four control bits. The gain control range of the PGA is 15 dB with 1 dB gain-steps. The total gain control range of two PGAs in series is 30 dB. The output swing of the signal of PGA is 600 mV p-p.

## 7. Experimental Measurement

**Figure 8:**
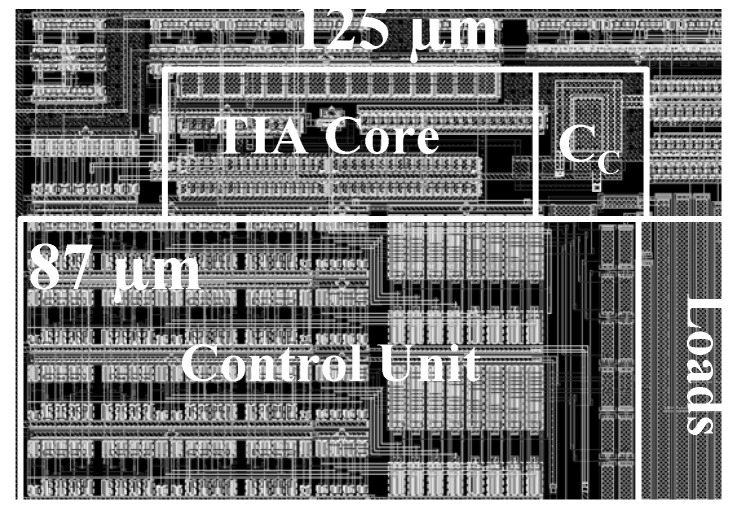
The top layout of the TIA and the area occupation of its sub-blocks.

**Figure 9:**
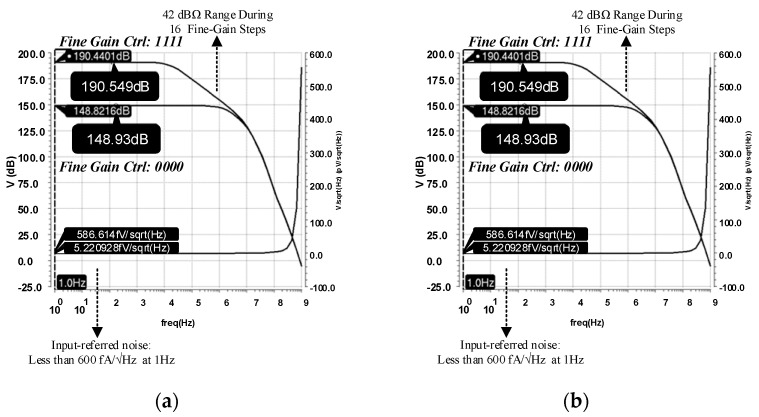
The simulation results of the (**a**) fine-gain and (**b**) course-gain modes.

**Figure 10:**
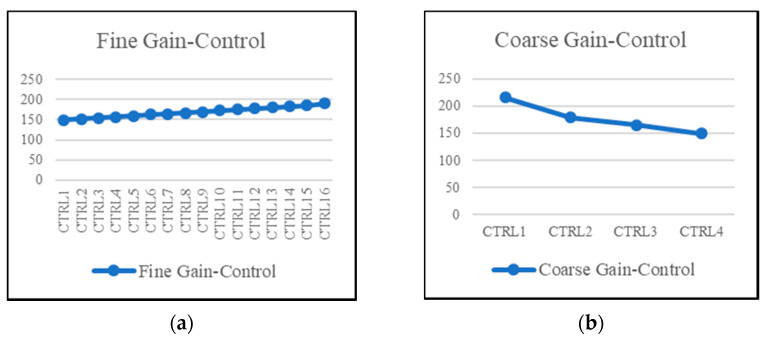
Gain of the TIA versus the (**a**) fine-gain controls and (**b**) the coarse-gain controls.

**Figure 11:**
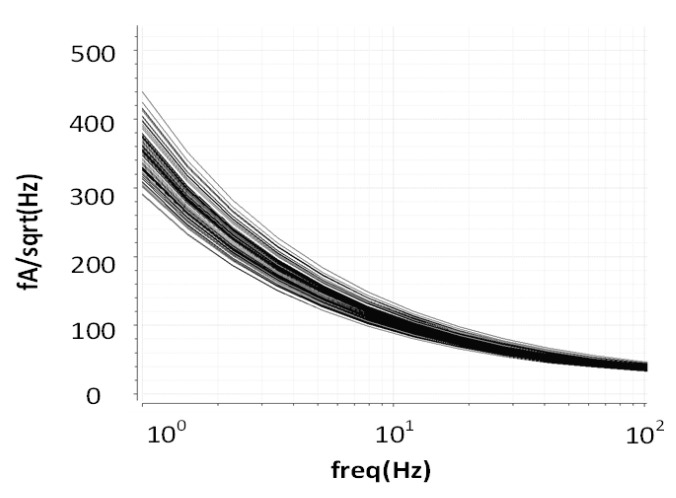
Monte-Carlo analysis for the total input-referred noise.

The chip of the proposed fine-dust detection system is fabricated in a 0.18 µm standard CMOS process. The die size of the chip is 1100 µm × 1100 µm. To emphasize the low area occupation of the TIA, its top layout is shown in Figure 8. Post-layout simulations show a dynamic range of 42 dB with 16 gain-steps for the fine-gain steps, as shown in Figure 9a. Furthermore, coarse-gain-steps with a 67 dB dynamic range are shown in Figure 9b. Figure 10a,b show the gain of the TIA versus fine- and coarse-gain controls, respectively. The input-referred noise of the TIA, in all cases, is kept at less than 600 fA/√Hz and goes as low as 27 fA/√Hz at 20 kHz. A Monte-Carlo analysis provides a total input-referred noise with a 150 fA/√Hz variation, as shown in Figure 11.

**Figure 12:**
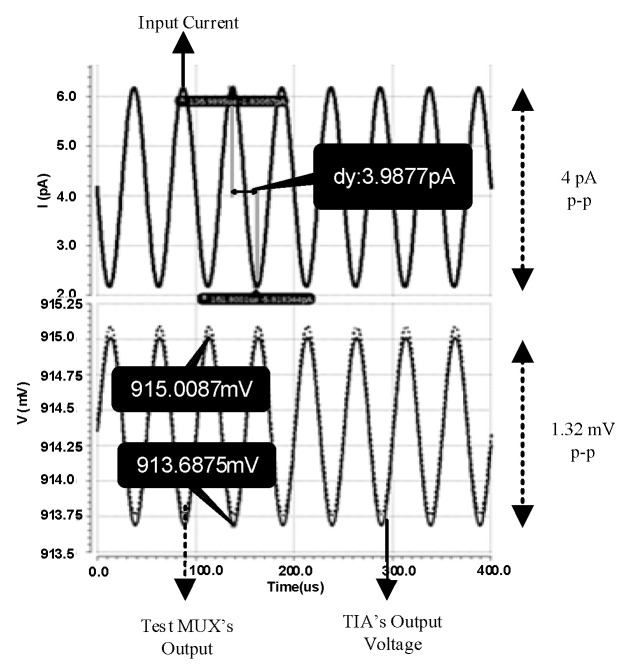
The transition simulation result for the minimum sensitivity of the TIA.

Post-layout simulations in transition show high sensitivity for the TIA, which is capable of amplifying signals as small as 4 pA resulting in a 1.32 mV output. This is shown in Figure 12. In this term, the transition gain is obtained around 170 dBΩ. For all the simulations, the environmental effects of the chip, including the pad and the model of the bonding wire of the chip, are considered.

**Figure 13:**
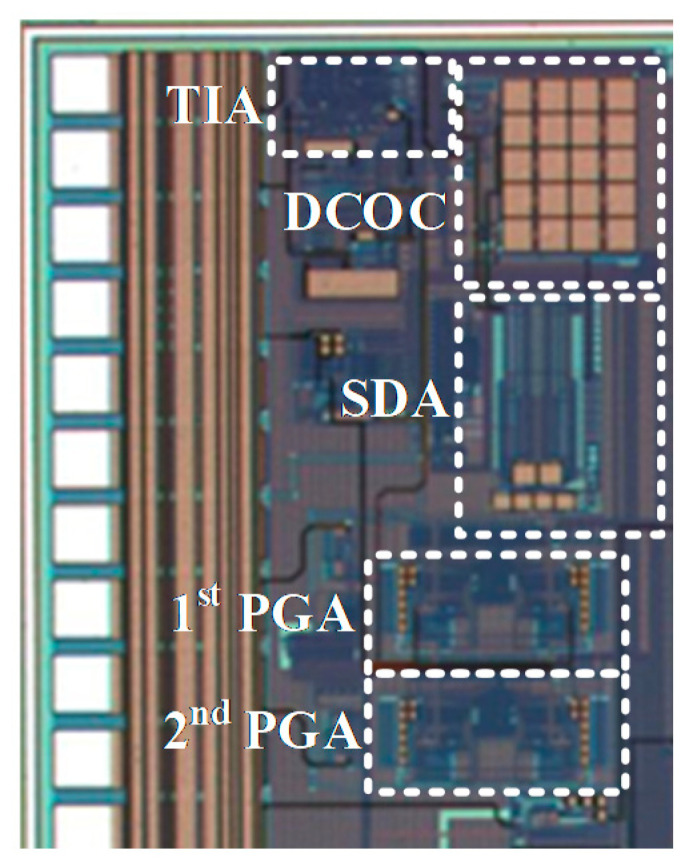
The chip-micrograph of the fine-dust detection system, including the proposed analog front-end and TIA.

**Figure 14:**
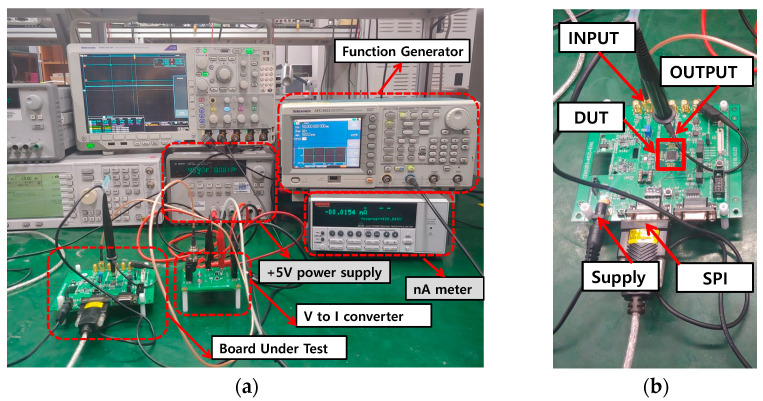
The measurement (**a**) equipment and (**b**) setup.

The chip-micrograph of the analog front-end is shown in Figure 13. A fine-dust detection sensor module is installed on the test board. The structure of the module consists of a laser beam and a photodiode that detects the variation of observed light from the laser beam when dust particles are passing through the module. The measurement setup of the proposed analog front-end is shown in Figure 14, which is formed by an oscilloscope, power supply generator, pulse controller, PC interfaces, the Device-Under-Test (DUT) board, and a voltage-to-current (V-I) converter to provide input currents for the TIA. The V-I converter is used to convert the input pulse from the pulse generator to the input current signals in the range of nano-amperes. 

**Figure 15:**
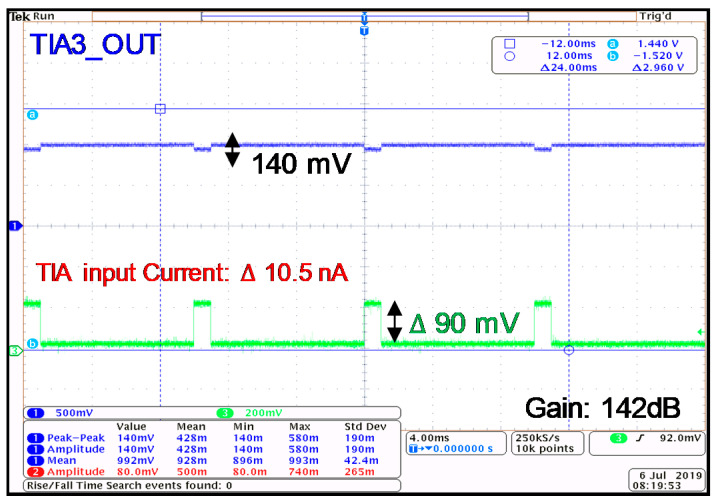
The measured maximum gain for the TIA.

**Figure 16:**
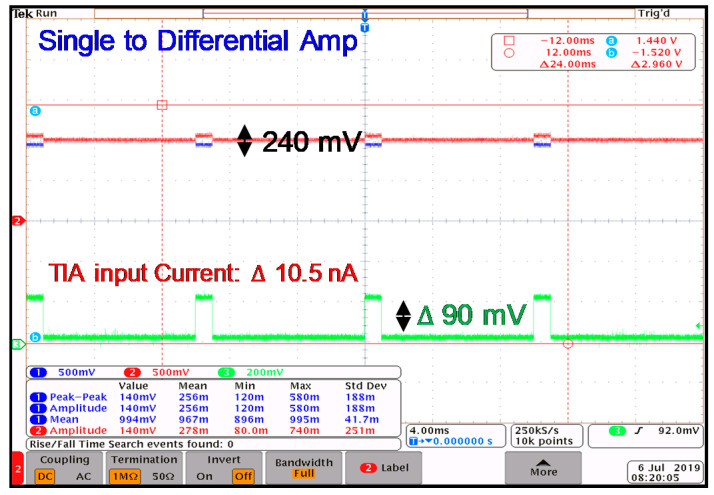
The measured output of the SDA.

**Figure 17:**
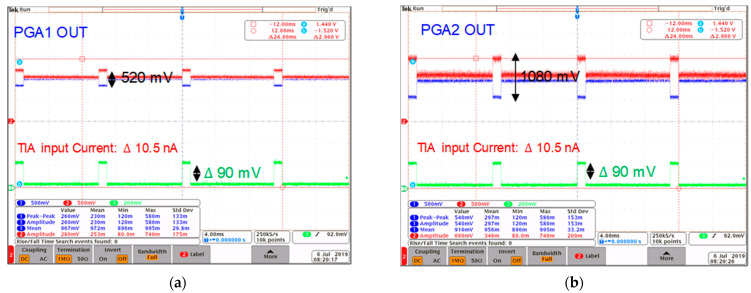
The measured output of the (**a**) PGA1 and (**b**) PGA2.

The measured gain of the TIA in the highest gain-mode is shown in Figure 15, which shows a 142 dB gain for the TIA when the V-I converter is delivering a 10.5 nA input signal to the TIA. Next, the output signal of every stage of the analog front-end is measured. Figure 16 shows the measured signal to a differential amplifier, providing the differential signal at the output. The amplified signal after the PGA1 and PGA2 is illustrated in Figure 17a,b. The performance of the TIA is compared with similar works in Table 1.

The comparison table shows the dual-mode gain adjustability of the TIA and the analog-front end as the superiority of this work over the similar works. The proposed TIA consumes less current, while the area occupied is kept small. The maximum gain of the TIA is a high value, which provides higher sensitivity for the proposed analog front-end. The dynamic range of the proposed analog front-end is obtained as 77 dB due to the gain adjustability of the building blocks.

## 8. Conclusions

This paper presents an analog front-end for a fine-dust detection system with a dual-mode ultra-low noise TIA is proposed. The TIA and analog front-end are fabricated in a 0.18 µm standard CMOS technology. Post-simulation results show a low IRN, as low as 600 fA/√Hz in low frequencies, and less than 27 fA/√Hz at 20 kHz, a minimum detectable current signal of 4 pA, and has a power consumption of 2.71 mW. Experimental results of the fabricated chip, by measuring the full path of the analog signal conditioning path, show a maximum gain of 142 dB for the TIA. The Single-to-Differential Amplifier delivers a differential waveform with a unit gain. The PGA1 and PGA2 show a maximum gain of 6.7 dB and 6.3 dB, respectively. The full-path analog front-end shows a wide dynamic range up to 77 dB in the measurement results.

## Figures and Tables

**Table 1:** Comparison table over various TIA structures.

Parameters	[10]	[11]	[13]	[14]	This Work
Technology (CMOS)	0.18 µm	0.35 µm	0.35 µm	0.18 µm	0.18 µm
Supply (V)	3.3	1.8	N/A	1.8	3.3
Topology	Common-Mode Amplifier	Shunt-feedback amplifier	3-stage push-pull inverters	Dual-mode CMOS feed-forward	Adjustable-Gain 3-stage Push-pull inverters
C_PD_ (pF)	2	0.5	10	0.5	1
Gain (dBΩ)	106	65	107.3	76	142
Application	LADAR	Ethernet	SEM	LADAR	Bio
Bandwidth	50 MHz	1.1 GHz	12.5 MHz	720 MHz	20 kHz
Maximum IRN pA/√Hz	1.52	10.9	3.54	N/A	0.03
Coarse-Gain Steps Dynamic Range (dB)	-	-	-	-	67
Fine-Gain Steps Dynamic Range (dB)	-	-	-	-	42
Power Consumption (mW)	8	6.2	60	20.7	2.71
Area $\left(µm^{2}\right)$	283.8 k	NA	15.9 k	117.5 k	10.9 k
